# CTDA: an accurate and efficient cherry tomato detection algorithm in complex environments

**DOI:** 10.3389/fpls.2025.1492110

**Published:** 2025-03-13

**Authors:** Zhi Liang, Caihong Zhang, Zhonglong Lin, Guoqiang Wang, Xiaojuan Li, Xiangjun Zou

**Affiliations:** ^1^ School of Mechanical Engineering, Xinjiang University, Urumqi, China; ^2^ Institute of Agricultural Mechanization, Xinjiang Academy of Agricultural Sciences, Urumqi, Xinjiang, China

**Keywords:** picking robot, cherry tomato detection, deep learning, YOLO, multi-scale feature fusion

## Abstract

**Introduction:**

In the natural harvesting conditions of cherry tomatoes, the robotic vision for harvesting faces challenges such as lighting, overlapping, and occlusion among various environmental factors. To ensure accuracy and efficiency in detecting cherry tomatoes in complex environments, the study proposes a precise, realtime, and robust target detection algorithm: the CTDA model, to support robotic harvesting operations in unstructured environments.

**Methods:**

The model, based on YOLOv8, introduces a lightweight downsampling method to restructure the backbone network, incorporating adaptive weights and receptive field spatial characteristics to ensure that low-dimensional small target features are not completely lost. By using softpool to replace maxpool in SPPF, a new SPPFS is constructed, achieving efficient feature utilization and richer multi-scale feature fusion. Additionally, by incorporating a dynamic head driven by the attention mechanism, the recognition precision of cherry tomatoes in complex scenarios is enhanced through more effective feature capture across different scales.

**Results:**

CTDA demonstrates good adaptability and robustness in complex scenarios. Its detection accuracy reaches 94.3%, with recall and average precision of 91.5% and 95.3%, respectively, while achieving a mAP@0.5:0.95 of 76.5% and an FPS of 154.1 frames per second. Compared to YOLOv8, it improves mAP by 2.9% while maintaining detection speed, with a model size of 6.7M.

**Discussion:**

Experimental results validate the effectiveness of the CTDA model in cherry tomato detection under complex environments. While improving detection accuracy, the model also enhances adaptability to lighting variations, occlusion, and dense small target scenarios, and can be deployed on edge devices for rapid detection, providing strong support for automated cherry tomato picking.

## 1 Introduction

The cherry tomato is a small tomato known for its rich nutritional value and wide-spread market demand, making it one of the important economic crops worldwide (Chen et al., 2021). Harvesting cherry tomatoes is a critical process in their agricultural production. Current harvesting methods rely primarily on manual labor, which presents problems such as high labor costs, low harvesting efficiency, and other related challenges. In addition, these methods cannot meet the demands of large-scale production, which hinders the sustainable development of the cherry tomato industry (Ishii et al., 2021; Montoya-Cavero et al., 2022). In recent years, with the advancement of agricultural mechanization and automation technologies, robotic harvesting has gradually become a viable alternative. The accuracy, adaptability, and real-time capabilities of robotic vision systems are the primary technological supports for reliable robotic harvesting, and they also determine harvesting efficiency (Magalhães et al., 2022; Li Y. et al., 2024). Computer vision has a wide range of applications in various fields (Wang H. et al., 2023; Tang et al., 2024), such as robotic navigation, 3D imaging (Li et al., 2023; Li H. et al., 2024), and remote sensing (Jamal Jumaah et al., 2024), which are crucial for enhancing the efficiency and accuracy of agricultural tasks like fruit harvesting. Currently, most cherry tomatoes are cultivated in greenhouses. Compared to traditional open-field cultivation, greenhouse cultivation adopts relatively standardized practices optimized for mechanized harvesting, such as uniform plant spacing, consistent plant height, and structured plant arrangement, providing an agronomic basis for robotic harvesting. However, under greenhouse conditions, variations in lighting, occlusions caused by the clustering growth of fruits, the small size of the picking targets, and the complexity of the background (including background interference from the intermingling growth of plant branches and leaves, and changes in the color distribution between fruits and leaves) due to the coupling effects of multiple factors, increase the difficulty of visual detection, impacting the precise identification and localization of fruits. Variations in the height between individual plants, irregularity in the arrangement of leaves, the uneven distribution of fruits, and partial or complete occlusion between fruits and leaves are unstructured characteristics that pose numerous challenges to the visual detection of picking robots (Zhang et al., 2024).

Traditional target detection methods based on image processing are classic approaches to fruit detection, which require extracting target features and learning to classify and recognize them. Morphological and color-based analysis methods have been widely applied to fruit detection. Septiarini et al. (2022) proposed a tomato image segmentation method, applying K-means clustering for ROI detection, performing RGB to HSV color space conversion in preprocessing, and using the Canny operator for edge detection, successfully achieving tomato feature extraction and detection. Li J. et al. (2024) proposed single and dual-mode image demodulation, brightness correction, and image segmentation algorithms, utilizing fast average filtering based on integral images to enhance the contrast between rotten areas and fruit backgrounds. Their approach significantly improved the early detection of rotten navel oranges, achieving a recognition accuracy of 97.5% using a two-phase spiral phase transform (SPT) combined with contrast adjustment and watershed segmentation. Feature extraction and classification methods have been widely explored to improve recognition accuracy. Bai et al. (2023) proposed a tomato recognition method integrating HOG, LBP, and color histogram algorithms. By concatenating shape, texture, and color feature vectors into a combined feature vector and processing it using a Support Vector Machine (SVM), the model achieved 100% accuracy under ideal conditions, with a processing time of less than one second. Liu et al. (2019) introduced a mature tomato detection algorithm combining HOG features with an SVM classifier, using a coarse-to-fine scanning approach. The model was further refined using false color removal (FCR) and non-maximum suppression (NMS), achieving a recall rate of 90.00%, a precision of 94.41%, and an F1 score of 92.15%. Chaivivatrakul and Dailey (2014) proposed a plant green fruit detection technique based on texture analysis, employing interest point feature extraction, descriptor calculation, SVM classification, candidate fruit point mapping, morphological closure, and fruit region extraction. The model achieved detection rates of 85% and 100% for single images of pineapple and bitter melon, respectively. Traditional image recognition techniques often rely on manual feature design, which achieves fruit recognition in specific scenes, but lack an understanding of the overall semantics of the image and are not well adapted to complex and changing unstructured environments (Qi et al., 2022).

Compared to traditional image processing methods, deep learning employs deep neural networks to automatically extract hierarchical features, reducing reliance on manual feature engineering. Through large-scale data training, it optimizes feature representation, enhances robustness to noise and defects, and improves detection stability (Banerjee et al., 2023; Kasani et al., 2023; Zeng et al., 2023). In fruit detection, deep learning models have been widely used due to their superior feature representation capabilities and increased detection accuracy over conventional image processing techniques (Chen S. et al., 2023; Meng et al., 2023; Tang et al., 2023b). Lawal (2021) proposed the YOLO-Tomato model for detecting tomatoes under complex environmental conditions by applying the LWYS method with spatial pyramid pooling as well as Mish activation function and integrating the dense architecture into YOLOv3. The mAP reaches more than 98% on small resolution datasets and the detection time is less than 50ms. Zheng et al. (2022) developed an enhanced method for recognizing cherry tomatoes by improving the YOLOx model. This approach integrates an attention mechanism within the dense network’s backbone to enhance overall recognition performance. While these methods enhance detection performance by incorporating additional modules or modifying existing components, they also result in a larger and more complex model. This increased complexity poses challenges for deployment and utilization on edge devices. Therefore, to solve the challenges associated with implementing complex models on edge devices for robotic harvesting, researchers are paying more attention to balancing accuracy and model complexity while enhancing model performance. For instance, Gao et al. (2024) proposed LACTA, a lightweight high-precision sage fruit detection algorithm with a model size of only 2.88 M, which can be better deployed to selective harvesting robots. Yang et al. (2023) proposed an automatic tomato detection method based on an improved YOLOv8s model. The mAP of the enhanced model was increased by 1.5%, and the model size was significantly reduced from 22 M to 16 M. At the same time, a detection speed of 138.8 FPS was achieved, which is a better balance between the model size and detection accuracy. However, most of the lightweight algorithms proposed currently target tomatoes with distinct close-range features, are set in relatively simple background environments and do not consider the environmental impacts experienced by robots during actual harvesting operations, often resulting in somewhat singular data samples (Zhang et al., 2023). Therefore, further research into cherry tomato detection algorithms in actual working environments, ensuring that deployment on edge devices still maintains satisfactory real-time performance and accuracy, remains a highly challenging issue.

To realize accurate and efficient cherry tomato recognition in complex environments, this paper proposes an accurate lightweight, real-time, and efficient saint fruit detection algorithm. Firstly, to enhance the detection model’s adaptability to the diversity of fruit features, lighting conditions, and background environments, this study employs a multi-source dataset augmentation strategy, selectively expanding the original dataset and establishing targeted datasets. Secondly, to further optimize and balance the detection efficiency and the capability to extract small target features under complex lighting conditions, the backbone network of the YOLOv8 model is reconstructed. The LAWDarknet53 network is introduced to replace the CBSDarknet53, allowing the model to retain more details while reducing redundant computations when extracting image features from shallow to deep layers. Considering the issues of occlusions, overlaps, and density that occur during the actual harvesting process, the SPPS network is proposed to better capture subtle feature changes caused by environmental variations. The introduction of a dynamic head detection head focuses on capturing valuable details, enhancing the model’s understanding and detection accuracy in complex environments. This algorithm adapts well to unstructured environments under natural conditions, possessing good generalization and robustness, capable of being deployed on edge devices to efficiently and effectively complete detection tasks while ensuring performance.

## 2 Materials and methods

### 2.1 Data acquisition

**Figure 1:**
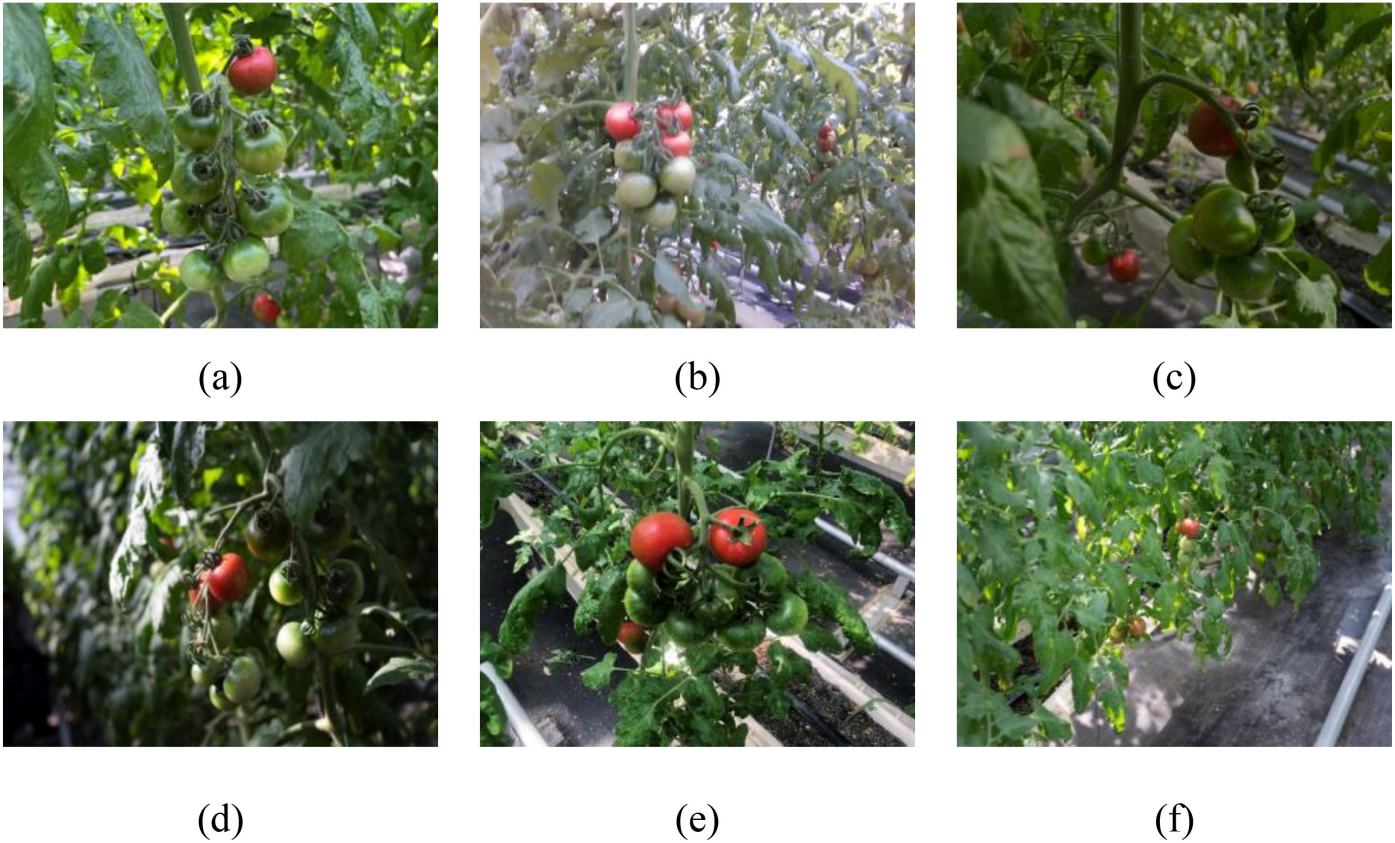
Images of cherry tomatoes captured in a complex greenhouse environment under various conditions: **(a)** natural lighting, **(b)** intense lighting, **(c)** dim lighting, **(d)** shaded areas, **(e)** overlapping clusters, and **(f)** small targets positioned at a relatively greater distance.

Data on cherry tomatoes were collected at the Changji Agricultural Expo Park planting base in Xinjiang. To ensure the consistency of the growing environment and the quality of the fruit, the planting base uses a uniform vertical planting system. In this study, data on cherry tomatoes was collected using a handheld portable camera with a resolution of 1920 × 1080. To ensure data diversity and broad coverage, images were collected under various lighting and occlusion conditions to simulate different actual planting environments, such as direct sunlight, shadows, fruit overlap, and occlusions. During the image collection process, the study captured images from multiple angles, including frontal, overhead, oblique, and upward angles, to capture features such as the shape, color, and texture of the cherry tomatoes, and conducted image collection at close, medium, and long distances to obtain fruit images at different scales. During the data collection period, the cherry tomatoes were in the ripening stage, with some of the fruits fully matured and meeting harvesting standards. A total of 2500 images of both mature and immature cherry tomatoes were collected, as shown in **Figure 1**.

### 2.2 Data preprocessing

**Figure 2:**
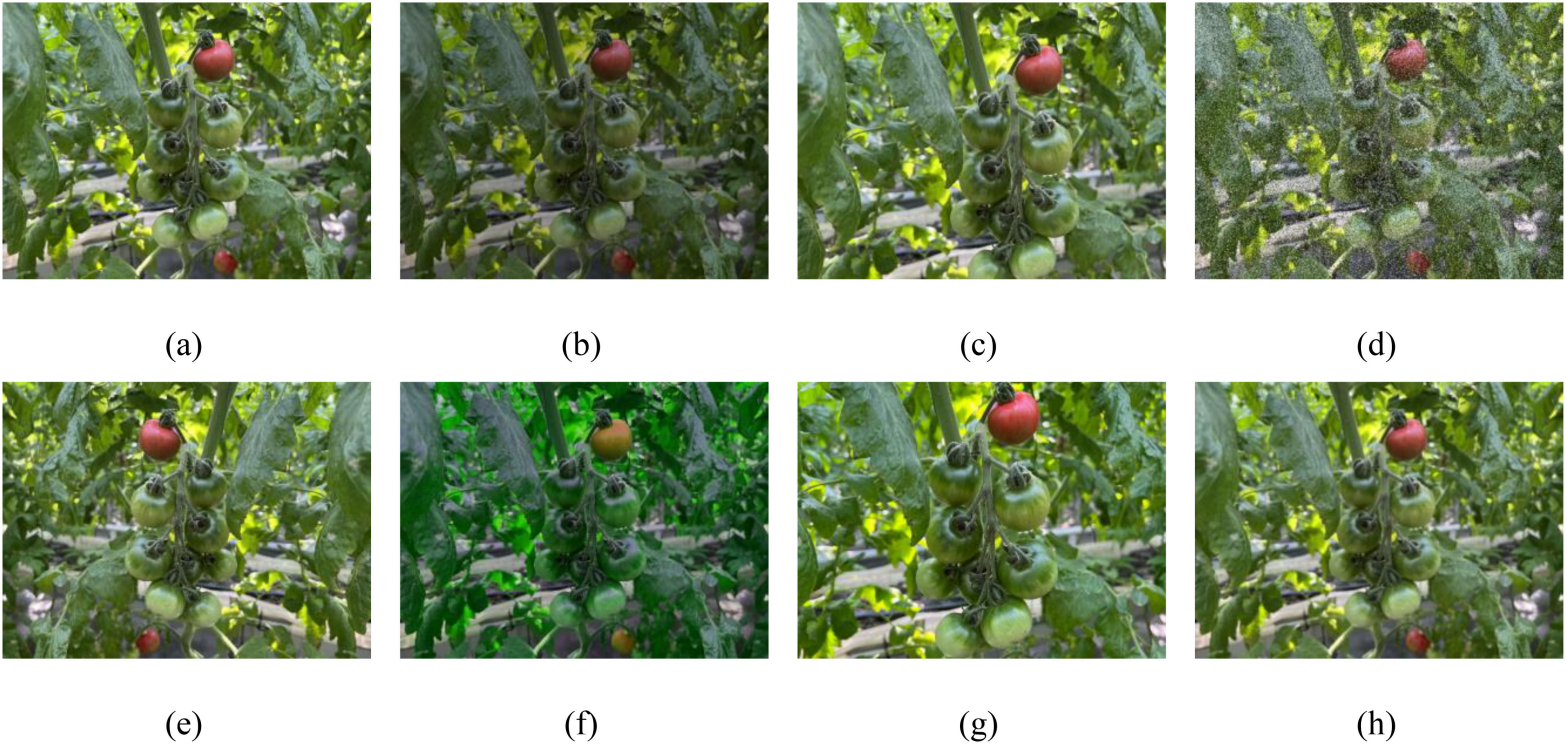
The original data is expanded using several techniques for data augmentation: **(a)** original data, **(b)** brightness, **(c)** translation, **(d)** noise, **(e)** horizontal flipping, **(f)** HSV, **(g)** scaling, **(h)** blurring, and their random combinations.

By increasing the amount and variety of data, data augmentation can simulate different planting environments, including different lighting and occlusion conditions, as well as different shooting angles and distances, improving the robustness of the algorithm and the model’s ability to generalize (Tang et al., 2023a). This study used a combination of offline and online data augmentation to selectively expand the original dataset; offline data augmentation is a preprocessing method applied to the original dataset before training, involving random combinations of brightness adjustment, rotation, translation, and noise to expand the dataset. Considering that excessive offline augmentation might introduce too much noise or inconsistency, potentially degrading model performance, only 500 new training samples are expanded using offline methods. Online data augmentation is the real-time enhancement of the original data during model training, which enhances model diversity while reducing storage resource requirements. During training, techniques such as noise, HSV adjustments, random rotations, scaling, and perspective transformations are used to enhance the training samples, with each method being applied with a 1% probability. It also turns off data augmentation in the last 10 epochs, allowing it to focus on learning from the original data, optimizing and complicating the details of the features (Ge et al., 2021). The effects of the enhancement are shown in **Figure 2**.

In the data labeling process, the study used the labeling tool to process the cherry tomato dataset “https://github.com/HumanSignal/labelimg”. During the data labeling process, the smallest enclosing rectangle of the cherry tomato was used as the true detection frame to minimize the interference of background information with the true detection frame. The samples were categorized into two groups: “Immature,” representing unripe cherry tomatoes, and “Mature,” indicating ripe cherry tomatoes. **Table 1** provides detailed information about the dataset. During the model training process, the training set used 80% of the dataset, the validation set used 10%, and the test set used 10%, guaranteeing that the test set is created using only the original photos and does not include any enhanced images.

**Table 1:** Differences between the offline enhanced dataset and the original dataset.

Category	Parameter		Training	Validation	Test	Total
Original dataset	Number of images		2000	250	250	2500
	Instances	Immature	7261	1052	974	
		mature	6790	956	851	
		All	14051	2008	1825	17884
Augmented dataset	Number of images		2400	300	300	3000
	Instances	Immature	8834	1321	1241	
		mature	8286	1196	1142	
		All	17120	2517	2383	22020

### 2.3 CTDA model

**Figure 3:**
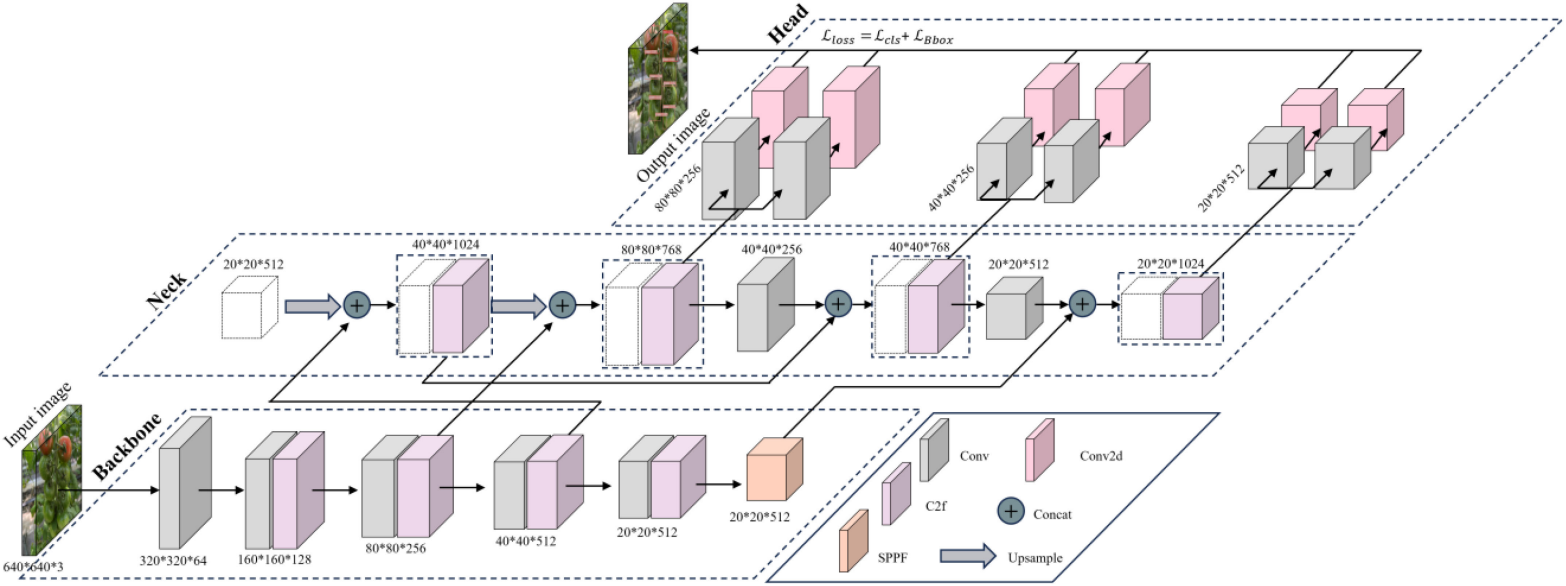
YOLOv8 model architecture diagram.

YOLO (You Only Look Once) was released in 2015 as a fast, accurate, and widely applied real-time object detection algorithm. The basic idea is to treat the target detection task as a regression problem and make predictions in an end-to-end manner. YOLOv8, the latest real-time object detector released in early 2023 as part of the YOLO series, establishes new technical standards for instance segmentation and object detection “https://github.com/ultralytics/ultralytics”. **Figure 3** illustrates the architectural model of YOLOv8, which includes a backbone network, neck, and detection head. Compared to previous generations, the backbone employs CBSDarknet53 for four times subsampling to extract features, utilizing SPPF for multi-scale feature extraction, which improves the model’s ability to identify targets of varying sizes by capturing object and scene information at multiple scales. The neck uses FPN-PANet to merge and aggregate feature maps from different levels for a more global and semantically rich feature representation. The head section uses a decoupled head that separates classification and regression tasks, which effectively reduces the number of model parameters and computational complexity, improving model generalization and robustness.

**Figure 4:**
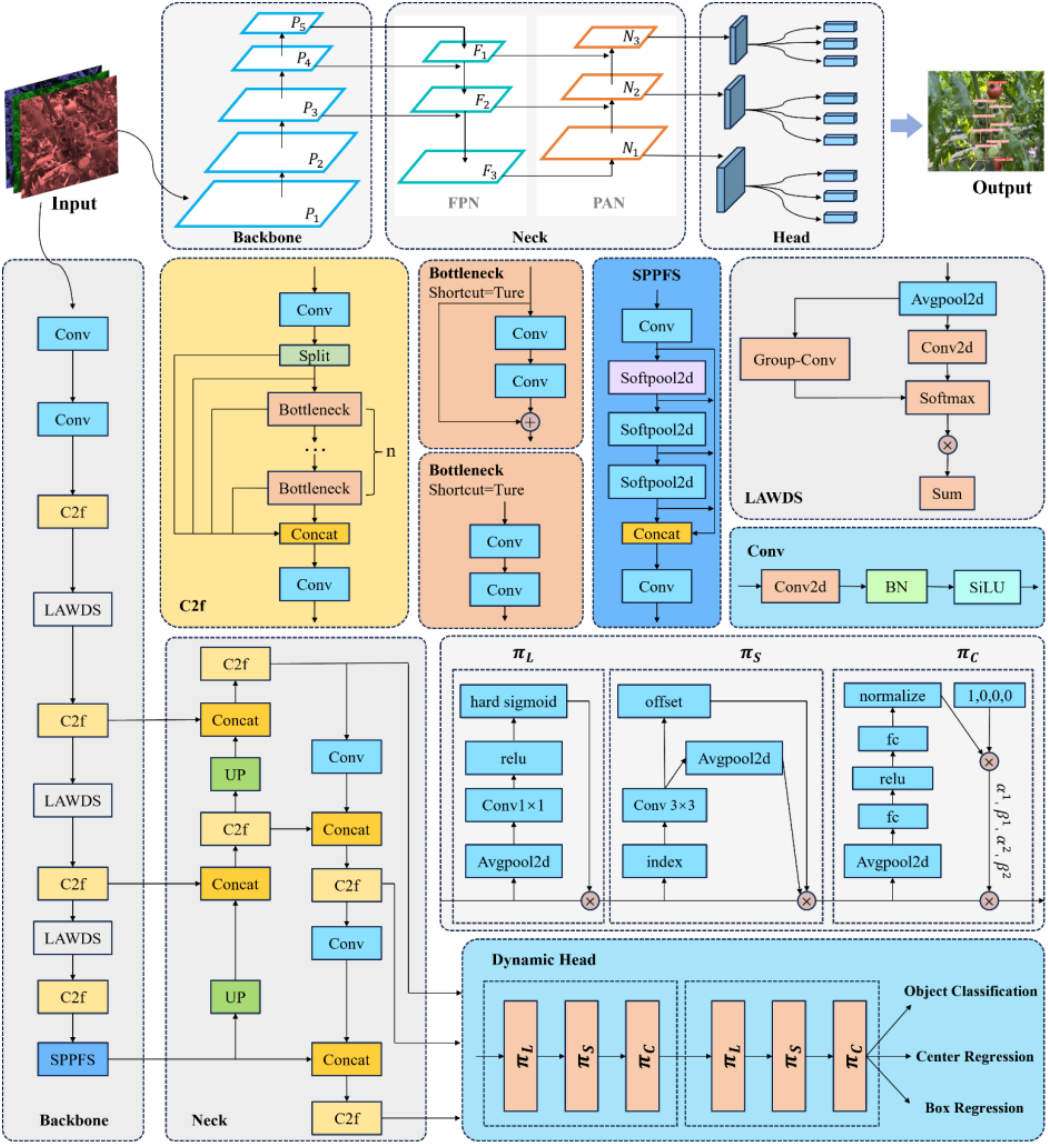
CTDA model structure diagram.

CBSDarknet53 experiences feature information loss, particularly with clustering, occlusion, and distant small targets, which reduces the model’s detection accuracy. SPPF, which builds residual networks through maxpool at different levels, increases the risk of feature information loss during downsampling, thereby raising the likelihood of missing detections of partially occluded cherry tomatoes. Decoupled head, focusing on separating the classification and localization tasks, tends to overlook the relationships between targets and local context information, potentially causing false or missed detections in scenarios with dense targets or occlusions. This article improves the structure of the YOLO8 network model and introduces a new network model to address these issues. Firstly, a new downsampling method, LAWDS, incorporates adaptive weights and receptive field spatial features to preserve important features better and enhance feature representation; it maintains spatial information continuity and avoids disrupting spatial relationships between adjacent pixels. Secondly, softpool replaces maxpool in SPPF; while maxpool activates features by selecting the maximum value, which is simple and efficient, it may lose important information. Softpool uses a weighted sum of softmax activations within the kernel area to optimize activation downsampling, preserving more background information and feature details, thus enhancing feature representation. Finally, the study introduces a dynamic head with attention mechanisms, using a unified attention mechanism for scale perception, spatial awareness, and task awareness within a single structure to enhance object detection performance, effectively improving the representational capability of the detection head. The model structure is shown in **Figure 4**.

#### 2.3.1 LAWDarknet53

In natural harvesting conditions, cherry tomatoes to be harvested by robots may be at a distance, presenting fewer features in images, it makes it particularly important to improve the model’s ability to detect small, distant targets. Additionally, real-world production involves issues such as bright or dim lighting, necessitating improvements in the model’s adaptability to different lighting conditions to ensure detection accuracy. In the entire detection network model, the backbone network is a crucial component for feature extraction; its performance directly affects the model’s ability to detect and locate targets. Therefore, improving the feature extraction capability of the backbone network is necessary to adapt to the diversity of cherry tomatoes at different distances, sizes, and lighting conditions.

In this application context, CBSDarknet53 exhibits certain limitations; it uses the CBS module for feature extraction and fixed convolution for downsampling, where convolution operations depend on common parameters and are insensitive to changes in position that cause variations in information. Different lighting conditions alter the features of cherry tomato images, and the CBS module fails to effectively adjust its feature extraction strategy, leading to the loss of crucial features. Furthermore, fixed convolution sampling can miss some small target features, and the convolution operations might disrupt spatial relationships between adjacent pixels, resulting in discontinuities in spatial information. To address these issues, the research introduces a new downsampling method called light adaptive weight downsampling (LAWDS). LAWDS incorporates adaptive weights and receptive field spatial features, better preserving essential features and enhancing the representation of features for distant small targets. The formula for its calculation is as shown in Equation 1:


(1) $$x_{\text{output}}=\sum_{i=1}^{4}(\text{Conv}{\left(x\right)}_{i}\cdot \text{Softmax}{\left(\text{AvgPool}2\text{d}(x)\cdot \text{Conv}(x)\right)}_{i})$$

Where $x_{\text{output}}$ is the output of the module, $\text{Conv}{\left(x\right)}_{i}$ represents the *i*th feature map obtained through the downsampling convolution operation, and $\text{Softmax}{\left(\text{AvgPool}2\text{d}(x)\cdot \text{Conv}(x)\right)}_{i}$ represents the *i*th attention map channel processed by the softmax function.

**Figure 5:**
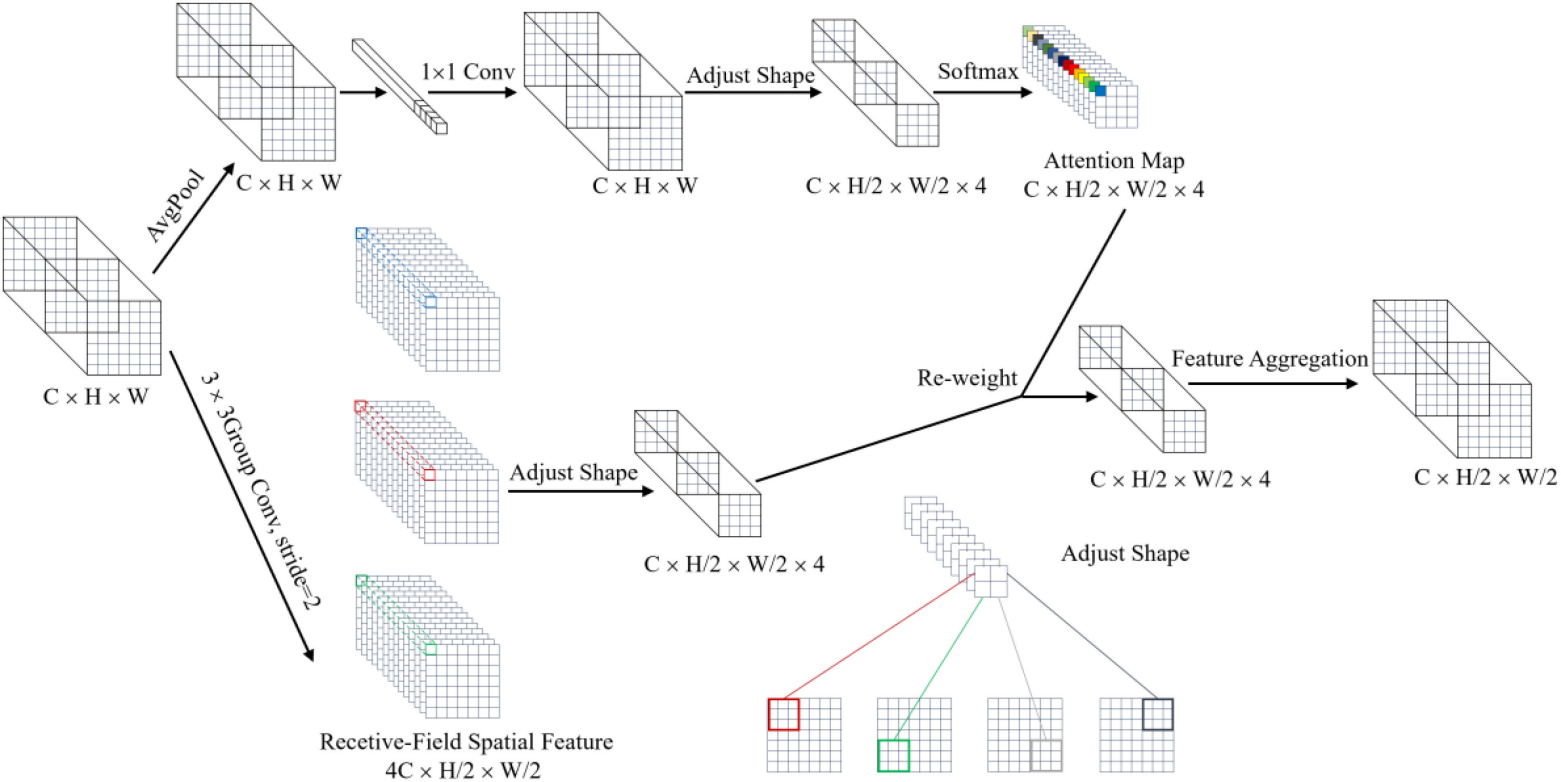
LAWDS structure diagram.

The LAWDS module initially employs average pooling operations to extract local features and gather global information. It then utilizes 1 × 1 convolution for inter-channel information exchange and feature transformation to further enhance the feature map’s expressive capacity. To improve the model’s focus on crucial features, a softmax function normalizes the attention map. Additionally, small targets, which typically have smaller sizes and lower pixel densities, can lose detailed information in traditional convolution operations. Compared to the Focus module in YOLOv5 (Zhao et al., 2022), grouped convolution in LAWDS offers similar effects but is more computationally efficient. This method splits the input feature map into several groups and performs independent convolution operations on each group, enabling quick and efficient extraction of receptive field spatial features, enhancing the perception of small targets, and reducing computational complexity. Finally, the LAWDS module implements weighted fusion and spatial weighting of features, thereby further improving the model’s focus on key features and increasing its performance and robustness. The structure of this module is shown in **Figure 5**.

#### 2.3.2 SPPFS

**Figure 6:**
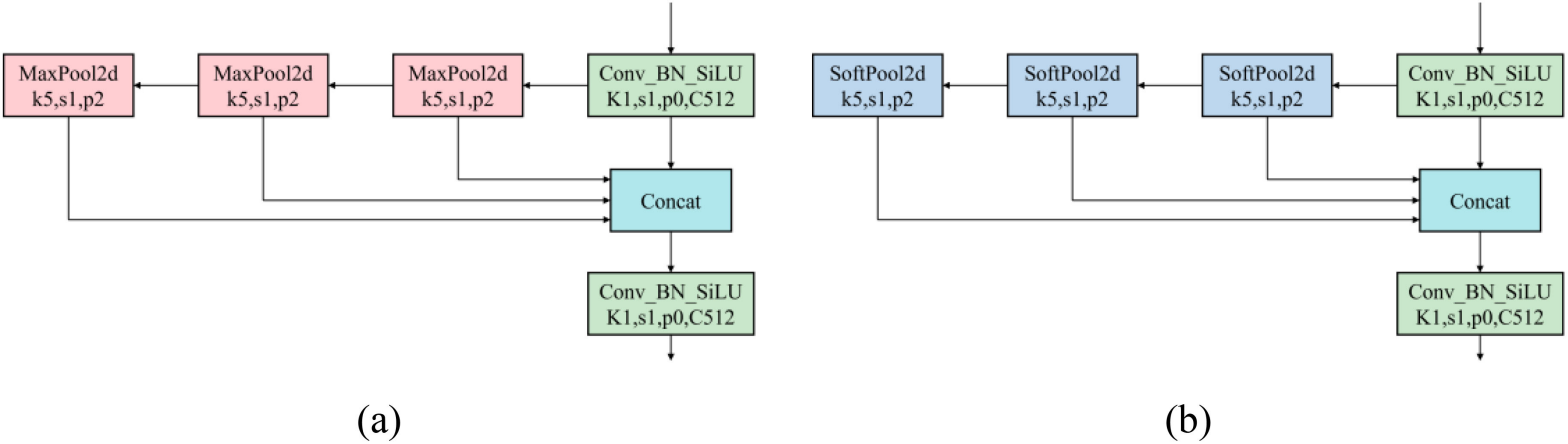
Using softpool to replace maxpool to build SPPFS: **(a)** SPPF structure diagram, **(b)** SPPFS structure diagram.

SPPF uses multiple pooling kernels to separate the most prominent feature information, and achieve optimal detection results by merging local and global features at the feature map level. However, in natural harvesting conditions, with fruits of different maturities varying in size and overlapping each other, the maxpool operation in SPPF leads to the loss of target feature information under dense occlusion, increasing the likelihood of missed detections of partially occluded cherry tomatoes. Therefore, the study proposes replacing the original SPPF network with the SPPFS network, as shown in **Figure 6**. This network uses softpool instead of maxpool, preserving more background information and feature details, enabling more efficient feature utilization and richer multi-scale feature fusion, reducing the limitations of maxpool in complex scenes, and better recognizing and differentiating tightly packed or partially occluded fruits, thereby enhancing overall detection performance (Stergiou et al., 2021). Furthermore, softpool, by more finely processing the activation maps, better captures and preserves subtle feature changes caused by these environmental variations. It strengthens the model’s resilience to environmental changes and increases the model’s accuracy in identifying cherry tomatoes, ensuring its stability and reliability in practical application scenarios.

**Figure 7:**
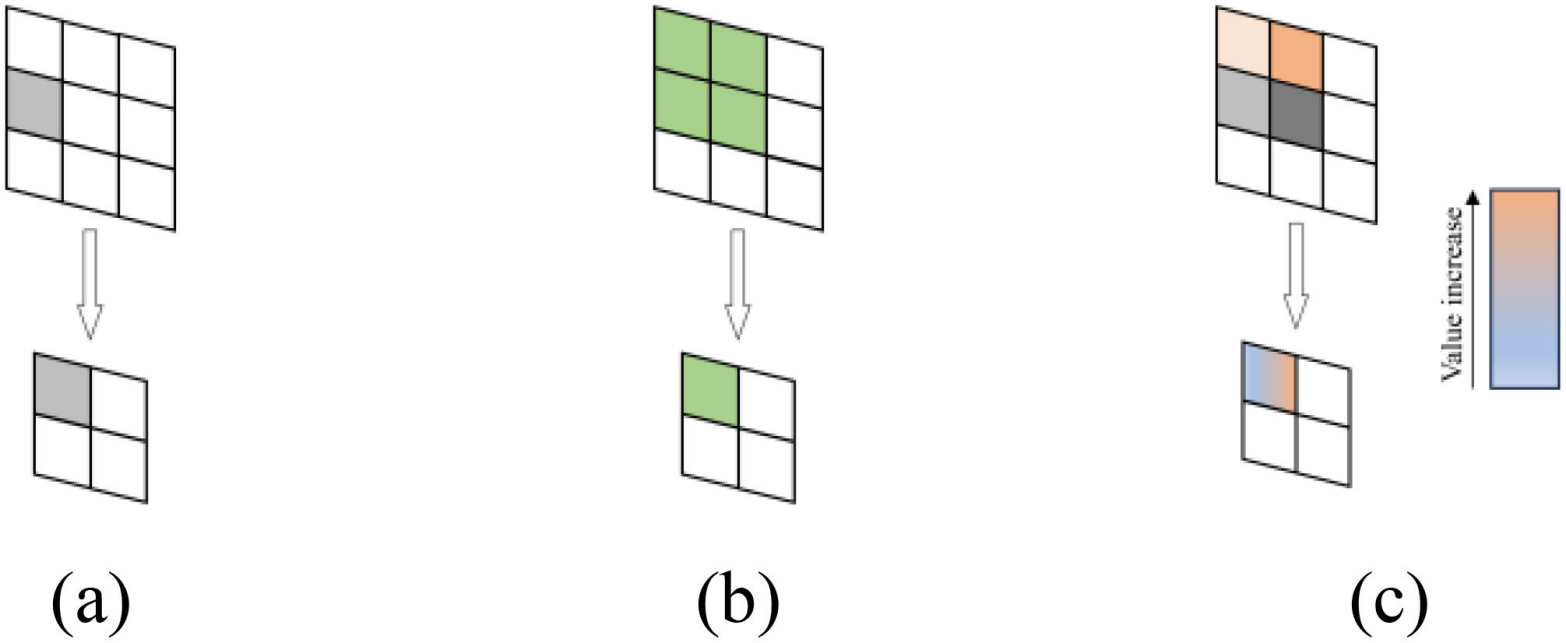
Schematic diagram of three pooling activation methods: **(a)** maxpool, **(b)** avgpool, **(c)** softpool.

In maxpool, discarding most activations carries the risk of losing important information. Conversely, in avgpool, the equal contribution of activations can significantly reduce the overall intensity of area features. Compared to maxpool and avgpool, softpool adopts an activation method within the kernel that uses softmax exponential weighting. This approach is designed to maintain the functionality of the pooling layer while minimizing information loss during the pooling process, as illustrated in **Figure 7**.

Softpool uses the maximum approximation of the activation region R. Each activation that has an index is given a weight that is calculated as the ratio of the natural index of that activation to the sum of the natural indices of all activations in the neighborhood R. The weight calculation formula is shown in Equation 2:


(2) $$wi=\frac{e^{ai}}{\sum_{j\in R}e^{aj}}$$

A nonlinear transformation uses the weights and the corresponding activation values. Larger activations are more common than smaller ones. Selecting the maximum value, which is the result of a standard summation of all weighted activations in the kernel neighborhood R, is not a balanced approach because most pooling operations are performed in high-dimensional feature spaces. Rather, it is better to highlight activations with greater effect. Its calculation formula is shown in Equation 3:


(3) $$\tilde{a}=\sum_{i\in R}w_{i}*a_{i}$$

#### 2.3.3 Dynamic head

YOLOv8 employs a decoupled head as its detection head, enabling the model to independently handle object classification and localization tasks. This design aims to increase processing speed and reduce interference between tasks, while simultaneously maintaining high efficiency and enhancing detection accuracy (Xiao et al., 2024). In the context of cherry tomato detection, where the fruits vary in size and density and may be clustered or partially obscured due to their growth characteristics, the decoupled head tends to overlook the relationships between targets and local context information in tasks involving multiple targets. This oversight can impact the detection effectiveness of cherry tomatoes in dense scenes.

Therefore, this study improves the head part of the original network with dynamic head, which introduces a scale-aware attention mechanism that more effectively captures target features, enabling precise detection of cherry tomatoes of varying scales, shapes, and densities (Dai et al., 2021). Especially in complex backgrounds with dense and highly overlapping targets, this mechanism aids in the model’s focus on key information, reducing the interference from background noise. The use of spatial awareness attention enhances the model’s comprehension of the spatial position of target objects, reducing errors in bounding box localization. Unlike the decoupled head, the dynamic head introduces a task-aware attention mechanism that dynamically adjusts its internal structure, including feature extraction and decision layers, based on the features of the input image. This dynamism allows the model to more flexibly handle various detection scenarios, enhancing the model’s generalization capability and accuracy.

The scale-aware attention module, which fuses features of different scales based on their semantic importance, is calculated as shown in Equations 4, 5:


(4) $$\pi_{L}(F)\cdot F=\sigma (f(\frac{1}{S\times C}\sum_{S,C}F))\cdot F,\;S=H\times W$$

(5) $$\left(x)=max(0,min(1,\frac{x+1}{2})\right)$$

where σ(x) is a hard sigmoid function, f(·) is a linear function approximated by 1 × 1 convolutional layers, and *H*, *W*, and *C* signify the height, width, and number of channels in the intermediate hierarchy, respectively. The feature tensor is represented by F .

The spatially-aware attention module focuses on the discriminative power of different spatial locations; given the high latitude of *S*, the module needs to be decoupled in two steps: first learning sparsification using variability convolution (Dai et al., 2017), and then aggregating features across levels at the same spatial location. Its calculation formula is shown in Equation 6:


(6) $$\pi_{S}(F)\cdot F=\frac{1}{L}\sum_{l=1}^{L}\sum_{k=1}^{K}w_{l,k}\cdot F(l;p_{k}+\text{Δ}p_{k};c)\cdot \text{Δ}m_{k}$$

Where *L* is the number of layers of the scaled feature pyramid, *k* is the number of sparsely sampled locations, $p_{k}+\text{Δ}p_{k}$ is the location shifted by the self-learned spatial offset $\text{Δ}p_{k}$ to focus on a discriminative region. $\text{Δ}m_{k}$ is the self-learned importance scalar at location $p_{k}$ , both learned from the mid-level input feature F .

The task-aware attention module facilitates collaborative learning and helps generalize different object representations, and it selects different tasks by dynamically turning feature channels on and off. Its calculation formula is shown in Equation 7:


(7) $$\pi_{C}(F)\cdot F=max(\alpha^{1}(F)\cdot F_{c}+\beta^{1}(F),\alpha^{2}(F)\cdot F_{c}+\beta^{2}(F))$$

where ${\left[\alpha^{1},\alpha^{2},\beta^{1},\beta^{2}\right]}^{T}=\theta (\cdot )$ is a hyperfunction that learns to adjust the activation thresholds, and $F_{c}$ is the feature slice of the *c*th channel. θ(·) is used in a manner similar to dynamic relu (Chen et al., 2020). To reduce dimensionality, it first performs global mean pooling on the L×S dimension. Then it uses two fully connected layers and a normalization layer, and finally it applies a shifted sigmoid function to normalize the output.

**Figure 8:**
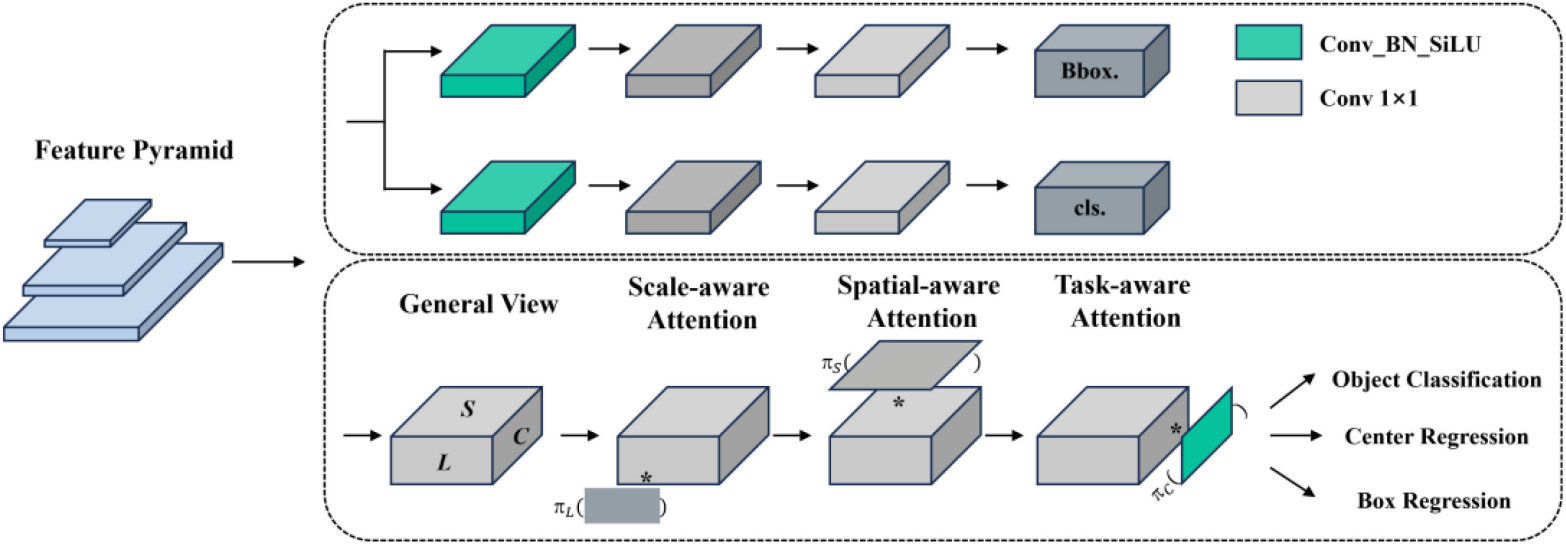
Schematic diagram of the decoupled head and dynamic head structures.

Dynamic head achieves the unification and synergistic effect of three types of attention mechanisms by sequentially applying scale-aware, spatial-aware, and task-aware attention modules. Additionally, YOLOv8 employs an anchor-free method, complicating the construction of specific task branches by attaching center or keypoint predictions to the classification or regression branches. In contrast, dynamic head simplifies the model structure and enables dynamic adjustments by merely attaching various types of predictions to the end of the head, as depicted in **Figure 8**.

### 2.4 Experimental environment and model evaluation indicators

All experiments in this study were conducted on a server equipped with an NVIDIA GeForce RTX 4090 GPU and an Intel(R) Xeon(R) Platinum 8375C CPU. The operating system was Ubuntu 20.04, and the experiments utilized CUDA version 11.8, python 3.8, and pytorch 2.0.0. The key hyperparameters used during the training process are outlined in **Table 2**. To verify the feasibility and effectiveness of the proposed improvements, experiments were carried out on the model. It is crucial to mention that these experiments were conducted using a baseline model, concentrating exclusively on validating the enhanced structure without incorporating pretrained weights.

**Table 2:** CTDA model training key hyperparameters.

Parameters	Values
Image Size	640×640
Epoch	200
Batch	16
Optimizer	SGD
Initial learning rate	0.01
Momentum	0.937
Weight decay	0.0005

The study evaluates the model performance of CTDA using precision (P), recall (R), mean average precision (mAP), F1 score, and GFLOPs. In object detection tasks, predictions are classified as positive samples when their intersection with the ground truth labels exceeds a certain threshold. Otherwise, they are identified as negative samples. The formulas for calculating precision and recall are shown in Equations 8, 9:


(8) $$\text{P}=\frac{TP}{TP+FP}\times 100\%$$

(9) $$\text{R}=\frac{TP}{TP+FN}\times 100\%$$

The mean Average Precision (mAP) represents the mean of the Average Precision (AP) values across multiple categories, and the AP formula is shown in Equation 10:


(10) $$\text{AP}=\int_{0}^{1}p(r)dr$$

mAP@0.5:0.95 refers to the average of the average precision calculated using different IoU thresholds between 0.5 and 0.95 in object detection.

The $F_{1}$ score is a reconciled average of precision and recall, which is used to comprehensively evaluate the model performance, ranging from 0 to 1, with higher values indicating better performance. Its calculation formula is shown in Equation 11:


(11) $$F_{1}=2\cdot \frac{Precision\cdot Recall}{Precision+Recall}$$

GFLOP refers to one billion floating point operations per second, which is used to evaluate the computational performance of the model.

## 3 Experimental results

### 3.1 The impact of augmented data on CTDA

Experiments were performed on three datasets to examine the effects of data augmentation techniques on CTDA performance: the original dataset, an offline-augmented dataset, and a dataset utilizing both offline and online augmentation. With the exception of the training dataset, the experimental conditions remained unchanged. The results presented in **Table 3** show that the offline augmented dataset increased precision by 0.7%, recall by 1.3%, and an average precision increase of 1.1% over the original dataset. The combined offline and online augmentation strategy further improved precision to 92.2%, recall to 86.7%, and mAP to 92.4%, indicating enhancements across all metrics. Thus, the data augmentation strategy combining offline and online approaches effectively improved the detection performance of CTDA.

**Table 3:** Data augmentation ablation experiment results.

original	Offline Enhancement	Online Enhancement	P(%)	R(%)	mAP(%)	mAP@0.5:0.95(%)
✓	×	×	91.9	85.2	90.9	69.5
✓	✓	×	92.6	86.5	92.0	69.9
✓	✓	✓	92.2	86.7	92.4	70.5

### 3.2 Comparison between different enhancement mechanisms of CTDA

**Figure 9:**
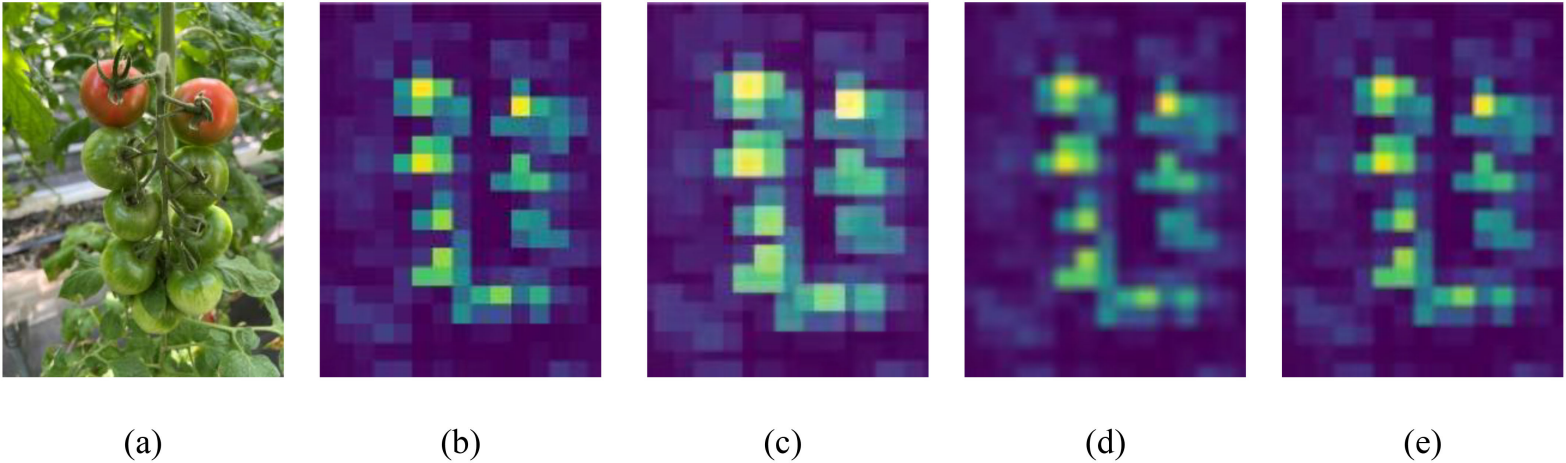
Visualization results of feature maps processed using different pooling mechanisms: **(a)** original image, **(b)** input feature map, **(c)** maxpool result, **(d)** avgpool result, **(e)** softpool result.

To improve the model’s ability to detect cherry tomatoes in occluded environments, softpool was used to replace maxpool in the SPPF, enhancing the detection of occluded sections. The effectiveness of softpool was assessed by applying maxpool, avgpool, and softpool treatments to the upper input feature maps of SPPF. As observed in **Figure 9**, maxpool led to the loss of critical features, which is particularly problematic for cherry tomatoes that inherently have fewer features, resulting in missed detections. While avgpool maintained important features, it reduced the intensity of the overall feature area, weakening of the model’s feature recognition ability. In comparison, softpool has significantly optimized this processing procedure, not only effectively preserving key features but also ensuring the overall intensity of the feature areas, which significantly increases the expressiveness of the features, and improves the model’s performance in the cherry tomato detection task.

**Figure 10:**
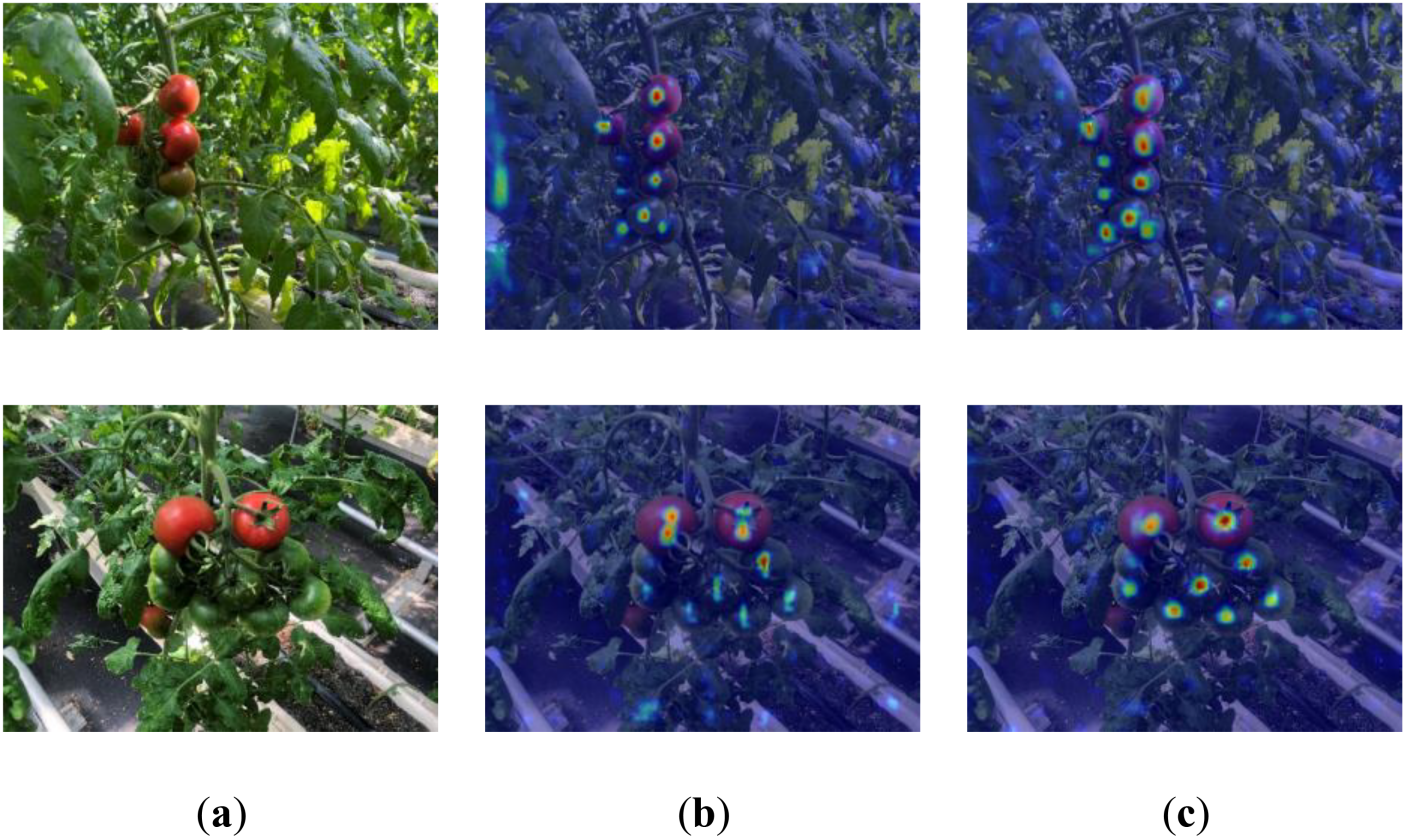
Visualization of different detection heads: **(a)** original image, **(b)** decoupled head detection effect, **(c)** dynamic head detection effect.

To improve the model’s detection ability for densely packed and highly overlapping cherry tomatoes in complex backgrounds, a dynamic head equipped with an attention mechanism was used to improve the head part of the original network. Both decoupled head and dynamic head were tested in dense cherry tomato scenes. **Figure 10** shows the heatmap generated using Grad-CAM, highlighting that CTDA primarily focuses on leaves and cherry tomatoes, with the background impacting detection. The study emphasizes distinguishing between foreground and background to focus solely on cherry tomatoes in the foreground. The heatmap indicates various detection heads’ differing attentiveness to dense cherry tomatoes. Dynamic head precisely targets these areas, reducing background interference. Experimental results show that CTDA based on dynamic head more effectively distinguishes and focuses on cherry tomatoes in dense areas, significantly improving foreground-background differentiation.

### 3.3 Ablation experiment

To further test the validity of the proposed strategies for improvement, this study incrementally tested the performance enhancements of the model. The testing process and results of the ablation experiment are shown in **Table 4**. Ablation testing showed that the B model, featuring the LAWDarknet53 structure, slightly exceeded baseline precision and recall, reducing the detection model size from 6.0 M to 5.4 M. Building on the B model, the C model incorporated the SPPFS network, significantly improving precision, recall, mAP@0.5, and mAP@0.5:0.95, with a slight increase in parameters. Integrating the dynamic head module into the CTDA model, which builds on the C model, resulted in a slight reduction in detection speed but notably enhanced precision, mAP, and mAP@0.5:0.95. Compared to the baseline model (A), the proposed CTDA model increased precision, recall, mAP@0.5, and mAP@0.5:0.95 by 2.1%, 5.2%, 2.9%, and 6.0%, respectively, achieving values of 94.3%, 91.5%, 95.3%, and 76.5%. Furthermore, the model size and FPS, at 6.7M and 154.1 respectively, only decreased by 1.1%, confirming that the model sustains high efficiency and real-time performance while notably improving accuracy. The results underscored the effectiveness of the improvement strategies, particularly in improving detection accuracy, where the LAWDS structure, SPPS component, and dynamic head module had significant impacts on computational parameters, recall, and precision, respectively.

**Table 4:** Model improvement ablation experiment results.

Model	Baseline	LAWDarknet53	SPPFS	Dyhead	P	R	mAP@0.5	mAP@0.5:0.95	Size/M	FPS
A	✓	×	×	×	92.2	86.7	92.4	70.5	6.0	155.8
B	✓	✓	×	×	92.8	88.3	93.5	74.9	5.4	154.9
C	✓	✓	✓	×	93.7	90.2	94.6	75.9	6.2	156.3
CTDA	✓	✓	✓	✓	94.3	91.5	95.3	76.5	6.7	154.1

### 3.4 CTDA network model training and testing

**Figure 11:**
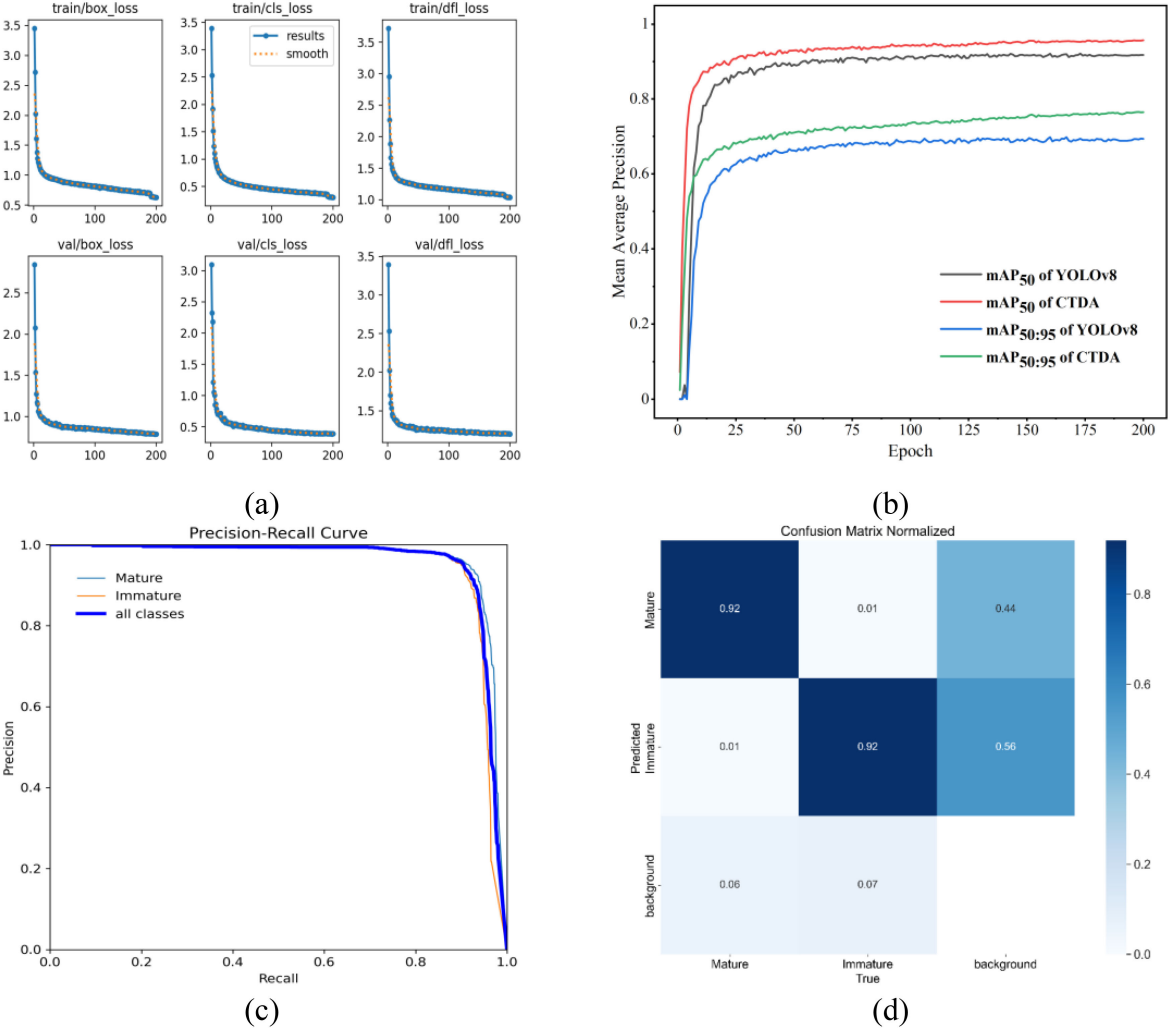
Training results for the CTDA model: **(a)** variations in loss during training, **(b)** comparison of average precision curves between CTDA and YOLOv8 during training at IoU thresholds of 0.5 and 0.5:0.95, **(c)** P-R curve, **(d)** confusion matrix.


The study conducted training of the CTDA model for 200 epochs on the enhanced dataset, with results depicted in **Figure 11**. YOLOv8’s loss computation includes classification loss ( VFL ) and regression loss ( CIoU loss plus Distribution Focal Loss ( DFL )), all weighted according to specific ratios. The formulas for these calculations are shown in Equations 12–15:

(12) $$VFL(p,q)=\{\begin{array}{cc} -q(q(\text{log}(p)+(1-q)\text{log}(1-p)) & q>0\\ -\alpha p^{\gamma }\text{log}(1-p) & q=0 \end{array}$$

(13) $$L_{\text{CIoU}}=1-\text{IoU}+\frac{\rho^{2}(\text{b},{\text{b}}^{\text{gt}})}{{\text{c}}^{2}}+\alpha v$$

(14) $$DFL(S_{i},S_{i+1})=-((y_{i+1}-y)\text{log}(S_{i})+(y-y_{i})\text{log}(S_{i+1}))$$

(15) $$S_{i}=\frac{y_{i+1}-y}{y_{y+1}-y_{i}},\;S_{i+1}=\frac{y-y_{i}}{y_{y+1}-y_{i}}$$

where *q* stands for the label; IoU for the intersection over union; b and ${\text{b}}^{\text{gt}}$ for the center points of the two rectangular boxes; *p* for the Euclidean distance between them; c for the diagonal distance of the enclosed region of the boxes; *v* for the consistency of their relative proportions; α for the weighting coefficient; *y* for the total distribution value; and *i* for the number of entries.

**Figure 11** displays the loss function curve, mAP curve, P-R curve, and confusion matrix of the CTDA model. According to **Figure 11a**, as training epochs rise, all three loss values progressively drop until stabilizing. **Figure 11b** shows significant improvements in mAP values for the CTDA model compared to the original YOLOv8 at IoU thresholds of 0.5 and 0.5:0.95. **Figure 11c** shows the precision-recall curves for Mature, Immature, and all categories during the training process. The Figure indicates that the area under the precision-recall curve for Mature is larger than that for Immature, indicating that the model performs better in identifying mature cherry tomatoes. This is because immature cherry tomatoes have a color similar to the plant, which interferes with their detection, whereas mature fruits have a clear contrast with the background environment, making them easier to accurately identify.

**Figure 11d** presents the confusion matrix for the CTDA model, with the vertical axis indicating the predicted labels and the horizontal axis displaying the actual labels. The color of each item represents the likelihood of that entry. Every category’s likelihood of being correctly classified is represented by the values along the major diagonal. It can be observed that both mature and immature cherry tomatoes have a classification probability of 92%. Values deviating from the main diagonal indicate model misclassifications. For this experimental result, the frequency of misclassifications is relatively low. Misclassifications mainly occur when the background is recognized as mature (44%) or immature (56%) cherry tomatoes.

### 3.5 Performance Test of CTDA

**Figure 12:**
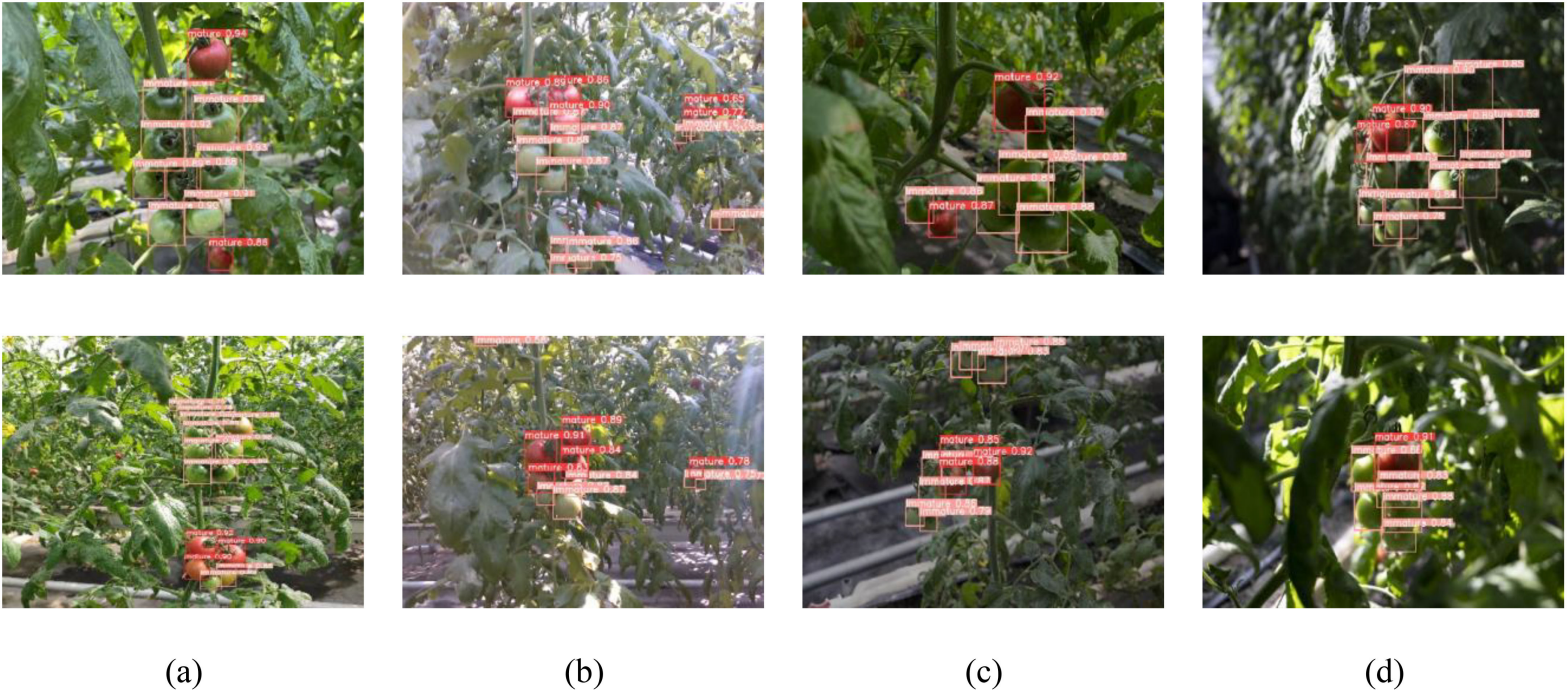
Effects of model detection in different illumination conditions: **(a)** natural lighting, **(b)** intense lighting, **(c)** dim lighting, **(d)** shaded areas.

The CTDA model’s performance under various lighting conditions, as depicted in **Figure 12**, includes natural lighting, intense lighting, dim lighting, and shaded areas. The detection results clearly show that the CTDA model can precisely identify mature and immature cherry tomatoes under these complex lighting conditions, highlighted with red and orange boxes respectively. This demonstrates the model’s high adaptability and robustness to complex lighting variations, essential for cherry tomato picking robots to efficiently and stably perform in unstructured natural environments. Additionally, detection experiments were conducted on cherry tomatoes at near and far distances under four lighting conditions, where the model typically captures larger, more detailed images at closer ranges. However, at greater distances, recognition becomes more challenging due to smaller target images, increased noise, and less distinct features. Thus, the proposed model effectively handles different scales of recognition, maintaining the ability to effectively recognize cherry tomatoes and accurately assess their maturity at long distances, with detection accuracy nearly equal to that of close-range detection.

**Figure 13:**
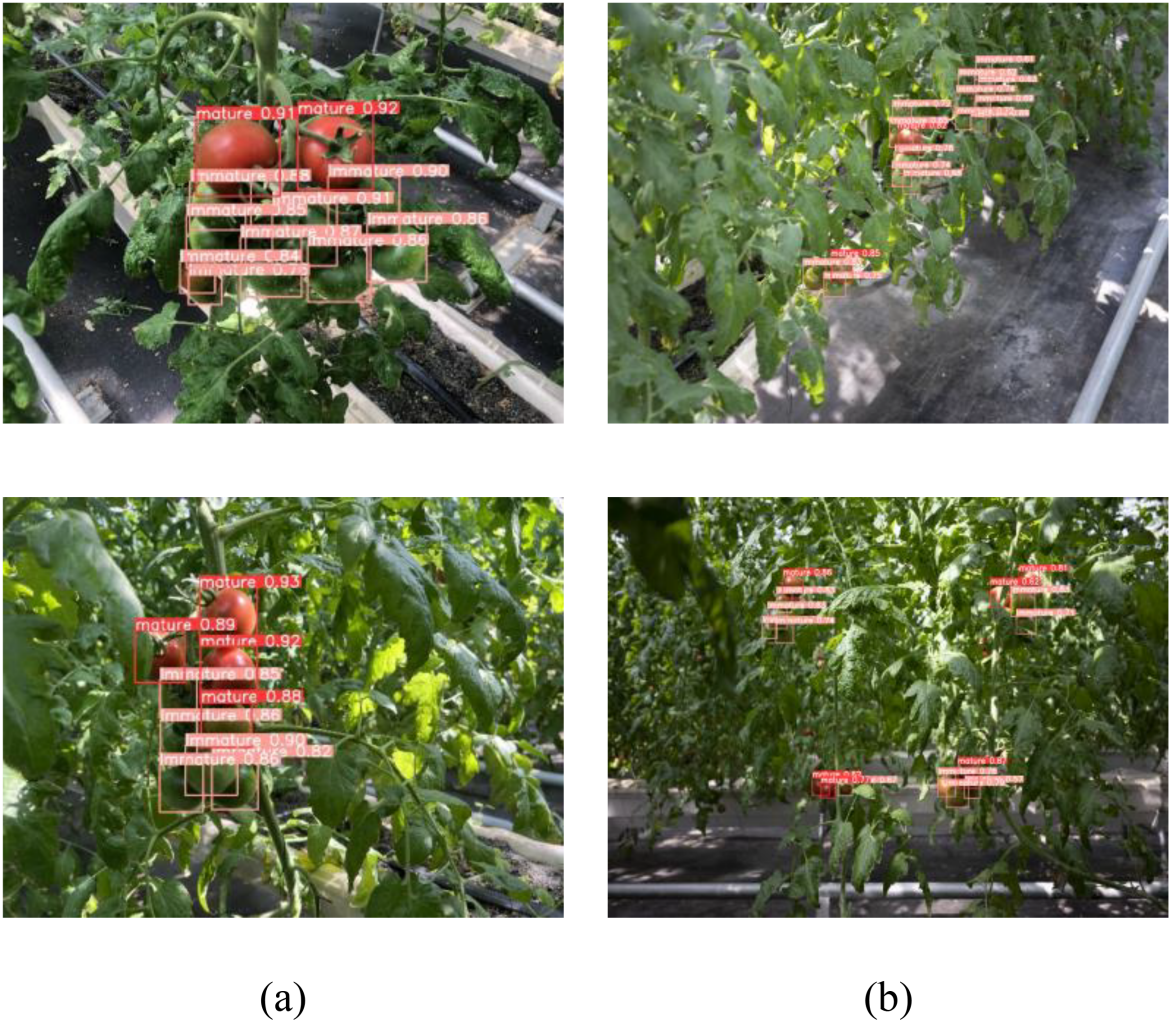
CTDA detection effect in different environments: **(a)** overlapping clusters, **(b)** small targets positioned at a relatively greater distance.

Due to cherry tomatoes growing in clusters and the limited spatial range of the robot’s visual sensor, there is a high occurrence of overlapping and small, distant targets during harvesting, which significantly impacts the model’s detection capabilities. The model is designed for efficient target detection in constrained operational spaces, ensuring it can differentiate between cherry tomatoes in the foreground and background, even when the targets are small or overlapping. **Figure 13** illustrates the CTDA model’s detection results in scenarios involving multiple overlaps and distant small targets, demonstrating the model’s ability to precisely detect and individually identify and locate each cherry tomato in overlapping situations. Furthermore, under other background disturbances like reflective mulching, the CTDA model continues to show outstanding detection performance, effectively distinguishing cherry tomatoes from complex backgrounds.

To evaluate the CTDA model’s detection capabilities in complex scenarios, this study developed a multi-scenario dataset, capturing images in greenhouses under various visual conditions including strong light, weak light, occlusion, and dense settings. The performance tests of the model included detecting both mature and immature cherry tomatoes, as well as determining the overall detection ability for all cherry tomatoes.

The study uses precision, recall, mAP and F1 score to evaluate the performance in different scenarios. As shown in **Table 5**, the model exhibited its best performance in detecting all cherry tomatoes under low light conditions, achieving the highest precision of 94.1% and an F1 score of 92.7. In the occlusion scenario, it achieved the highest recall rate of 92.9% and an mAP of 95.9%. In low-light and obscured conditions, the model performed better than in circumstances with high and dense light. Further analysis reveals that the model excels in detecting mature cherry tomatoes compared to immature ones. The CTDA model shows good detection ability in scenarios with strong light, low light, occlusion, and thick surroundings; nevertheless, strong light and dense circumstances significantly affect its performance.

**Table 5:** Detection results in complex scenarios.

Dataset	Classes	Instances	P(%)	R(%)	mAP(%)	F1(%)
Strong light	Immature	244	92.9	93.2	96.4	93.0
	mature	210	88.6	86.6	92.8	87.6
	Total	454	90.8	89.9	94.6	90.3
Weak light	Immature	271	95.9	87.6	94.8	91.6
	mature	224	92.3	95.2	96.4	93.7
	Total	495	94.1	91.4	95.6	92.7
Occlusion	Immature	406	87.3	92.2	95.1	89.7
	mature	336	94.3	93.6	96.7	93.9
	Total	742	90.8	92.9	95.9	91.8
Density	Immature	305	86.5	88.2	91.9	87.3
	mature	223	93.5	90.7	95.3	92.1
	Total	528	90.0	89.5	93.6	89.7

### 3.6 Robustness evaluation of CTDA in various contexts

**Figure 14:**
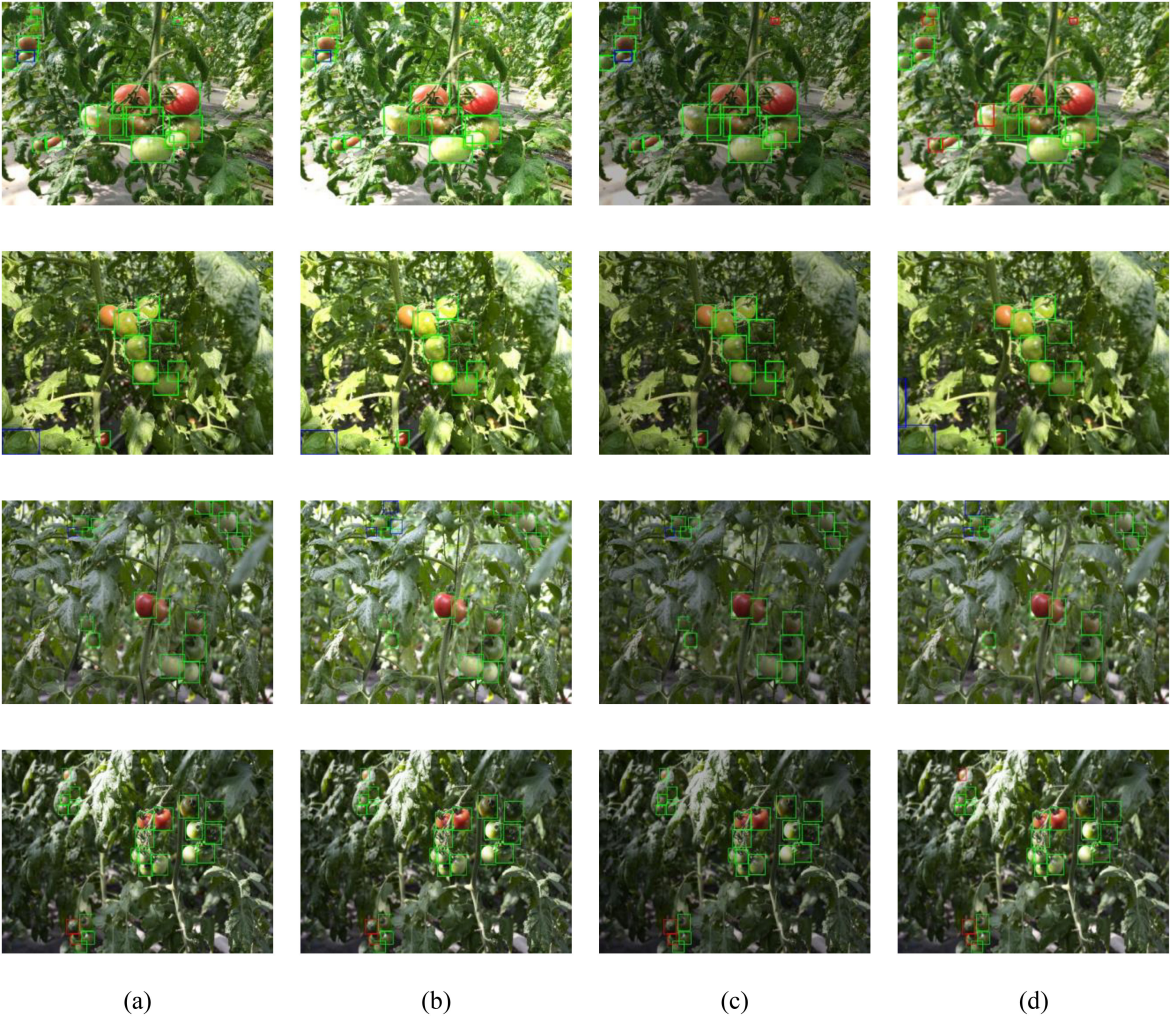
Visualization of cherry tomato detection outcomes in various settings: **(a)** under natural lighting, **(b)** intense lighting conditions, **(c)** low-light environments, and **(d)** blurred scenes. Green boxes represent accurate detections, blue boxes represent faulty detections, and red boxes indicate missing detections.

In the actual cherry tomato picking process, image quality can be severely impacted by environmental noises such as poor lighting (either insufficient or excessive) and blurring due to movement, thereby reducing the performance of target detection algorithms. To thoroughly evaluate the robustness and adaptability of the proposed CTDA model under different greenhouse conditions, four test datasets were created representing normal lighting, dim lighting, excessive lighting, and blurred images. These datasets, constructed by adjusting the brightness and adding blur to images from the normal lighting dataset, maintain consistency in image count, size, and annotations, with identical categorizations of the targets within each image. The model’s performance was visually assessed through visualization of the detection results in these scenarios, as illustrated in **Figure 14**, using green bounding boxes for correct detections, blue for incorrect ones, and red for misses. Despite some errors and omissions, the CTDA model generally excels in recognizing cherry tomato targets under different conditions. Because research divides detection targets into mature and immature categories, misclassification of mature as immature results in both a missed detection and an incorrect detection, leading to overlapping bounding boxes in the visual results.

As demonstrated in **Figure 14**, the CTDA model exhibits substantial stability and detection capabilities under variable lighting conditions, retaining high accuracy with minimal errors and misses under strong lighting, and showing even better performance in weak lighting. However, the model’s performance declines in blurred scenarios, with an increased rate of missed detections and a significant drop in detection accuracy, indicating a need for further enhancement in handling such conditions. The study observed that camera movement during the robotic picking process could cause image blurring due to external disturbances. Consequently, the research will focus on enhancing the model’s resistance to disturbances in blurred scenarios by augmenting the dataset with more images from such conditions, aiming to boost the model’s overall robustness.

### 3.7 Comparison of CTDA with the latest detection algorithms

In this study, the CTDA model is evaluated against both classical and state-of-the-art object detection models to further validate the effectiveness of the proposed algorithm, as presented in **Table 6**. These comparisons included models like Faster R-CNN (Ren et al., 2017), RetinaNet (Lin et al., 2017), EfficientDet (Tan et al., 2020), YOLOv5n, YOLOx-s (Ge et al., 2021), YOLOv7 (Wang C. Y. et al., 2023), Fasternet (Chen J. et al., 2023), Swin transformer (Liu et al., 2021) and RT-DETR (Zhao et al., 2023), encompassing high-precision, lightweight models, and representative detection algorithms. CTDA achieved the highest mAP of 95.3%, surpassing newly released models like YOLOv7 and Swin transformer by significantly reducing parameter counts by 91.1% and 88.1% respectively and increasing mAP by 3.4% and 4.9%. When compared to lightweight models like EfficientDet and YOLOv5n, CTDA showed a slight increase in parameters but far superior accuracy, outperforming them by 23.5% and 4.4% in mAP, respectively. Additionally, against models known for their fast detection speed, like Fasternet, CTDA not only improved mAP by 5.1% but also managed to reduce parameter count and increase FPS by 5.9% to 154.1, striking a good balance between speed and accuracy. This demonstrates CTDA’s exceptional overall performance, particularly in real-time capabilities, parameter efficiency, and accuracy, making it well-suited for deployment on edge devices with limited computing resources, thereby supporting efficient, precise, and real-time detection tasks.

**Table 6:** Comparative experiment of CTDA with other advanced models.

Model	P	R	mAP@0.5	mAP@0.5:0.95	GFLOPs	FPS
Faster R-CNN	78.8	80.7	84.1	58.5	369.7	22.6
RetinaNet	86.8	85.3	88.9	59.4	145.7	41.5
EfficientDet	78.7	67.1	71.8	48.9	4.7	23.8
YOLOv5n	92.8	84.1	90.9	69.4	7.1	144.6
YOLOx-s	93.3	86.4	88.7	68.4	26.76	81.2
YOLOv7	93.1	84.3	91.9	68.1	105.1	80.2
Fasternet	93.0	83.9	90.2	67.8	10.7	145.5
Swin transformer	92.6	83.2	90.4	67.7	79.1	46.6
RT-DETR	92.1	86.5	91.6	72.1	56.9	50.8
CTDA	94.3	91.5	95.3	76.5	9.4	154.1

## 4 Discussion

In unstructured environments, varying lighting conditions, complex backgrounds, and fruit overlap and occlusion pose challenges to the visual detection of picking robots. This study focuses on developing a detection algorithm tailored for use in picking robots. However, during the operation of the picking robot, there will be cherry tomato picking targets at a distance, which have fewer feature information in the image, making it difficult for cherry tomatoes to be effectively detected. Therefore, in response to these issues, the research proposes a precise, lightweight, and real-time efficient cherry tomato detection algorithm. By using LAWDS to reconstruct the backbone network, capturing more detailed features improves detection accuracy, making the model more effectively retain small target features. Secondly, the model introduces the SPPFS network, achieving more efficient feature utilization and richer multi-scale feature fusion, better identifying and distinguishing closely arranged or partially occluded fruits. The model applies the dynamic head detection head to more effectively capture target features, achieving accurate detection of cherry tomatoes of different scales, shapes, and densities. Additionally, the CTDA has significantly improved in accuracy, computational complexity, and detection speed while ensuring the model size, making it more suitable for deployment on resource-limited edge devices.

In model testing, an offline and online combined data augmentation strategy was utilized to selectively expand the original dataset, enhancing the model’s generalization capabilities. The study tested cherry tomato scenes under various lighting conditions, demonstrating the model’s adaptability to changes in lighting and its ability to accurately detect cherry tomatoes in scenarios involving overlap and distant small targets, effectively identifying each fruit even in overlapping states. The CTDA model also excelled in other background disturbances such as reflective mulching, effectively distinguishing foreground cherry tomatoes from complex backgrounds. A quantitative analysis showed minimal errors and missed detections under strong lighting, with better performance under weak lighting. However, blurred scenarios increased missed detections, significantly impacting accuracy, indicating room for improvement in the model’s handling of blurred images. External disturbances can cause image blurring during robotic harvesting in greenhouses, negatively impacting detection. Future work will explore optimizing the model to resist dynamic image blurring, possibly through attention mechanisms tailored for blurred target detection or image preprocessing techniques. Additionally, the current dataset primarily includes images of ripe and unripe cherry tomatoes, which limits the model’s comprehensive understanding of all growth stages. To improve the model’s detection capabilities and develop a more accurate automated picking system, the research will collect more data on cherry tomatoes of varying ripeness and growth stages. By analyzing the impact of different growth stages on model performance, more effective picking strategies can be devised, enhancing efficiency and reducing fruit loss due to incorrect picking.

In conclusion, while the CTDA model has its limitations, it has significantly contributed to improving cherry tomato detection technologies in greenhouse settings, providing essential technical support for the advancement of agricultural automation and intelligent development of harvesting robots. With continuous enhancements, this model is poised for wider future applications. Additionally, due to its adjustability and adaptability to various object features, the CTDA model’s framework and methodology could be adapted for other agricultural settings, particularly for fruit harvesting in complex environments, offering substantial technical support for robotic harvesting in unstructured settings. Further studies will also test the model across different crops and growing conditions to assess its utility and performance in a broader range of agricultural applications.

## 5 Conclusions

To enhance the detection capabilities for cherry tomatoes in complex environments, the study developed the CTDA model based on YOLOv8, tailored for unstructured settings. This model introduces a new downsampling method, LAWDS, to construct the LAWDarknet53 network, enhancing feature extraction capabilities. It also includes the SPPS network to improve feature fusion, addressing uneven detection issues in tomato occlusion scenarios. Additionally, the dynamic head with an attention mechanism was integrated to boost detection performance by harmonizing scale-aware, space-aware, and task-aware attention mechanisms within a single structure. The improved CTDA model achieved a 95.3% mAP, a 2.9% increase over the original, with significant improvements in recall and precision rates to 91.5% and 94.3%, respectively. To evaluate the effectiveness of the CTDA model in complex situations, datasets were generated that included strong, weak, occlusion, and density condition. The results showed accuracies of 94.8% and 95.1% in strong and weak illumination, respectively. The CTDA model demonstrates good stability under varying lighting conditions, but the miss rate increases in blurry scenes, affecting detection accuracy. The CTDA model was also compared with the latest detection networks, showing excellent performance in mAP, parameter count, and speed. Weighing 6.7M with a 95.3% mAP and 154.1 FPS, it meets the real-time detection requirements for cherry tomatoes in unstructured environments. Future research will integrate the CTDA model into cherry tomato harvesting robots to facilitate automated picking in greenhouses. Given mechanical vibrations can blur images during picking, reducing detection efficacy, ongoing research will aim to boost the model’s interference resistance, enhancing performance in disturbed environments and ensuring reliable visual support for cherry tomato harvesting robots.

## Data Availability

The raw data supporting the conclusions of this article will be made available by the authors, without undue reservation.
